# Development and Validation of Optical Methods for Zeta Potential Determination of Silica and Polystyrene Particles in Aqueous Suspensions

**DOI:** 10.3390/ma14020290

**Published:** 2021-01-08

**Authors:** Yannic Ramaye, Marta Dabrio, Gert Roebben, Vikram Kestens

**Affiliations:** European Commission, Joint Research Centre (JRC), 2440 Geel, Belgium; yannic.ramaye@ec.europa.eu (Y.R.); marta.dabrio@ec.europa.eu (M.D.); gert.roebben@ec.europa.eu (G.R.)

**Keywords:** electrophoretic light scattering, measurement uncertainty, method validation, particle tracking analysis, reference material, silica, zeta potential

## Abstract

Zeta potential is frequently used to examine the colloidal stability of particles and macromolecules in liquids. Recently, it has been suggested that zeta potential can also play an important role for grouping and read-across of nanoforms in a regulatory context. Although the measurement of zeta potential is well established, only little information is reported on key metrological principles such as validation and measurement uncertainties. This contribution presents the results of an in-house validation of the commonly used electrophoretic light scattering (ELS) and the relatively new particle tracking analysis (PTA) methods. The performance characteristics were assessed by analyzing silica and polystyrene reference materials. The ELS and PTA methods are robust and have particle mass working ranges of 0.003 mg/kg to 30 g/kg and 0.03 mg/kg to 1.5 mg/kg, respectively. Despite different measurement principles, both methods exhibit similar uncertainties for repeatability (2%), intermediate precision (3%) and trueness (4%). These results confirm that the developed methods can accurately measure the zeta potential of silica and polystyrene particles and can be transferred to other laboratories that analyze similar types of samples. If direct implementation is impossible, the elaborated methodologies may serve as a guide to help laboratories validating their own methods.

## 1. Introduction

Over the past decades, the use of engineered nanomaterials in consumer products has increased considerably [1,2,3]. As a consequence, concerns emerge about occupational exposure and potential adverse health effects [4,5]. In the European Union (EU), the main horizontal legislation protecting human health against harmful chemicals (the REACH Regulation (EC) No 1907/2006 [6]) was recently amended to address “nanoforms of substances” [7,8]. Nanoforms are identified as those materials that are considered “nanomaterials” under the European Commission’s Recommendation (2011/696/EU) on the definition of nanomaterial [9]. The amended Regulation obliges registrants of nanoforms to provide sufficient information and reliable data to demonstrate the safety of the nanoforms.

In supporting the implementation of legislation, significant advances have been made in the development and validation of new and existing methods for size analysis of (nano)particles [10,11,12,13,14,15,16]. As the nano-specific provisions in legislation carry a strong link to “particle size”, there has been less pressure on the validation of methods for the characterization of nanomaterial properties other than particle size so far. Method validation is a process used to quantify the performance of a method and to demonstrate its fitness-for-purpose [17].

To speed up the regulatory process, the risk assessment of groups of substances (including groups of nanomaterials), instead of individual substances, is expected to become more important. Grouping should facilitate the use of read-across of exposure and hazards [18,19]. For nanoforms, the concept of “sets of similar nanoforms” introduced in REACH is an additional type of grouping. The formation of sets of similar nanoforms is based on the physicochemical properties most relevant for the nanoforms’ behavior and reactivity. One of the critical material properties recommended to justify forming sets of nanoform is zeta potential (*ζ*-potential) [18,20].

Zeta potential, that is the electric potential in the interfacial double layer at the location of the slipping plane relative to a point in the bulk medium, is used as an indirect estimation of the surface charge density of a particle when it is in an electrolyte solution [21]. Therefore, zeta potential is an important measurand to investigate the repulsive interactions between colloidal particles and the tendency of agglomeration. As a rule of thumb, suspensions with an absolute mean zeta potential greater than 30 mV are considered colloidally stable [20]. It must be noted that zeta potential is not directly measurable but is related to electrophoretic mobility by the Henry equation [22]:

(1) $$\mu =\frac{2\varepsilon \zeta }{3\eta_{0}}f\left(\kappa a\right)$$

where *μ* is the average electrophoretic mobility of the particle, *ε* is the relative permittivity of the medium, *ζ* is the average zeta potential, *η*_0_ is the dynamic viscosity of the medium, *κ* is the reciprocal of the double layer thickness and *a* is the radius of the spherical particle.

The ratio of the particle radius to the electrical double layer thickness is given by the dimensionless parameter *κa*, which varies from 0 to ∞. For increasingly large values of *κa,* the Henry function (*f*(*κa*)) approaches values of 1.0 (Hückel model) and 1.5 (Smoluchowski model), respectively. The Smoluchowski approximation is only valid for aqueous media with moderate electrolyte concentrations (10^−3^ molar salt) and non-conducting spherical particles with a diameter > 100 nm [21].

A common technique for measuring the mean electrophoretic mobility of particles is electrophoretic light scattering (ELS) [23]. This cost-efficient technique owes its popularity to its ability to analyze samples within a short time (typical 3–5 min), while requiring only little sample preparation [24]. Existing alternative techniques determine electrophoretic mobility, for instance, by tracking individual particles using video microscopy (e.g., particle tracking analysis, PTA [23]) or by analyzing the colloid vibration current (e.g., electroacoustic methods [25]). The main advantage of ELS over PTA and electroacoustic methods is its ability to analyze samples of very different concentrations. On the other hand, PTA and electroacoustic methods require highly diluted samples (typically 10^7^–10^9^ particles/mL) and highly concentrated samples (>10 g/kg), respectively [14,25].

Although their popularity and the availability of documentary standards [22,23], the ELS and the PTA methods have yet been rarely validated. The authors are, to the best of their knowledge, aware of only one other published single-laboratory validation study conducted by Varenne et al. [26]. In that study, the performance of an ELS protocol was investigated for poly(isobutylcyanoacrylate) nanoparticles. The trueness of the results was assessed by analyzing a certified reference material (CRM) with a positive electrophoretic mobility (NIST SRM 1980 [27]) and a non-certified reference material with a negative zeta potential (Malvern DTS 1235 [28]). Given the fact that DTS 1235 is not a CRM, the researchers acknowledged that DTS 1235 was not ideal for estimating the uncertainty associated to the trueness of the ELS method when used for analyzing particles with a negative zeta potential.

Adequately validated methods are essential for accurate data and reliable assessment of product and regulatory compliance. Thus, in this study we aimed to (1) develop ELS and PTA methods to measure the zeta potential of polystyrene and silica particles in aqueous suspensions with equivalent diameters > 100 nm and negative surface charges, (2) validate the developed methodologies, and (3) estimate measurement uncertainty. The validation studies were designed to fulfill the requirements of ISO/IEC 17025 and recommendations of EURACHEM [29,30]. Measurement uncertainties were estimated according to ISO/IEC Guide 98-3 [31]. In the end, the validated methods will be used by the European Commission’s Joint Research Centre (EC-JRC) to support the production of new colloidal silica CRMs. In addition, the validated methods and the estimated measurement uncertainties can be transferred to routine laboratories that measure zeta potential of similar sample systems. If direct implementation is impossible, for instance due to different types of sample systems, our manuscript will serve as a practical guide to help laboratories demonstrating the fitness-for-purpose of their own methods.

## 2. Materials and Methods

### 2.1. Reference and Test Materials

Water-based suspensions of highly spherical polystyrene and near-spherical silica particles analyzed throughout the validation studies are listed in Table 1.

A non-certified reference material (RM), DTS 1235, was purchased from Malvern Panalytical (Worcestershire, UK). This material consists of carboxylated polystyrene particles in a proprietary buffer at a mass fraction of 0.2 mg/kg. DTS 1235, recommended by Malvern Panalytical for qualification tests of ELS instruments was analyzed both as-received and after dilution with the proprietary buffer whose exact composition was provided (under a non-disclosure agreement) by the manufacturer for the purpose of the validation study. For zeta potential experiments, dilution with the original dispersion medium is generally recommended as to avoid alteration of the electric double layer thickness due to a changing electrolyte concentration. The dilution factors applied were 2, 5, 10, 20 and 50 (PTA study) and 2, 4, 8, 16, 32, 64 and 100 (ELS study).

Two non-certified RMs with product codes A54091 and A54495 were kindly provided by Beckman Coulter, Inc. (Miami, FL, USA). The two materials, which are further identified as PS-A (A54091) and PS-B (A54495) are polystyrene particles dispersed in 10 mM NaCl at a mass fraction of 0.01 g/kg and 10 g/kg, respectively. PS-A was analyzed as-received. PS-B was diluted with 10 mM NaCl using 16 dilution factors resulting in samples with mass fractions in the range of 0.25 mg/kg to 5 g/kg. The 10 mM NaCl solution was prepared from a 1 M NaCl stock solution (Sigma-Aldrich NV, Bornem, Belgium), diluted with purified water (resistivity 18.2 MΩ cm at 25 °C).

A colloidal silica, Acesol WP4, was kindly received from Ace nanochem Co., Ltd. (Kyungsangbuk-Do, Korea). This material consists primarily of nominally 140 nm diameter particles. However, the presence of a fraction of particles with diameters smaller than 100 nm was demonstrated (results not shown). For such small particles, the Smoluchowski model is not valid, as the ratio of the particle radius to its double layer thickness (i.e., *κa*) becomes too small. Therefore, the fraction of nanoparticles (< 100 nm) was removed by means of tangential flow filtration (TFF) equipped with a mixed cellulose ester membrane of 0.1 μm cut-off. The resulting cleaned suspension (AWP4-A) had a particle mass fraction of 15 g/kg (as determined after 24 h of oven drying at 80 °C). A sub-sample of AWP4-A was further processed with TFF to obtain a more concentrated variant (AWP4-B) at 30 g/kg, which was used for dilution experiments during the ELS validation study. Dilutions were performed with 10 mM borate (Sigma-Aldrich NV, Bornem, Belgium).

Two CRMs, ERM-FD305 and ERM-FD306, certified for electrophoretic mobility and zeta potential, were obtained from EC-JRC (Geel, Belgium). Both materials have been produced from the same cleaned Acesol WP4 colloidal silica material. The CRMs differ in mass fraction, i.e., 1.5 g/kg (ERM-FD305) and 22 g/kg (ERM-FD306) [32,33].

In the present study, the CRMs ERM-FD305 and ERM-FD306 were used primarily to assess the trueness (i.e., performance parameter for bias or systematic error) of the zeta potential results. For ELS, both CRMs were analyzed as-received, i.e., without dilution. During the PTA validation study, ERM-FD305 was also analyzed to determine the method’s working range in terms of particle mass fraction. Before analysis, the CRM was diluted gravimetrically with 10 mM borate buffer at dilution factors ranging from 1000 to 50,000. The remaining RM (i.e., DTS 1235) and the test materials were used for precision experiments only. RMs and CRMs are characterized by the demonstrated stability and homogeneity with respect to their assigned property values. Furthermore, CRMs are characterized by a metrologically valid procedure allowing the assignment of certified values. The specified uncertainty of a certified value indicates its reliability as estimate of the true value [34].

### 2.2. Electrophoretic Light Scattering

Measurements of zeta potential by electrophoretic light scattering (ELS) were performed with a Malvern Zetasizer Nano ZS instrument (Malvern Panalytical, Worcestershire, UK) with implemented M3-PALS patented technology (see Appendix A). An overview of the applied measurement conditions is given in Table 2.

In ELS the particles move systematically (when they carry a net charge) according to an alternating electric field applied across a set of electrodes which are, depending on the type of sample cell, either embedded in the flow cell or submerged in the suspension (in case of a cuvette). In equilibrium conditions, the particles will move uniformly and at a constant velocity toward either the anode or the cathode, depending on the sign of their net charge. Because of the alternating motion of the particles, the frequency and phase of the scattered light, collected at an angle of 13°, will be different from that of the incident/reference light. This phenomenon, which is known as the Doppler effect, is used to determine the electrophoretic velocity, that corresponds to the electrophoretic mobility (*μ*):

(2) v=μE

(3) μ=∆ωλ04πEnsin(θ2)sin(θ2+ξ)

where *v* is the electrophoretic velocity, *E* is the electric field strength, *n* is the refractive index of the medium, Δ*w* is the Doppler frequency shift, *λ*_0_ is the wavelength of the laser light (in vacuum), *θ* is the angle between the incident light and the scattered light and *ξ* is the angle between the scattered light and the orientation of the electric field.

### 2.3. Particle Tracking Analysis by Video Microscopy

Measurements of zeta potential by video microscopy were performed with a NanoSight NS500 particle tracking analysis (PTA) instrument (Malvern Panalytical, Worcestershire, UK). An overview of the applied measurement conditions is given in Table 2.

The PTA instrument combines an optical microscope with a scientific complementary metal oxide semiconductor (CMOS)-based camera to visualize and record videos of scattered light from the suspended particles. By applying an electric field across the sample channel, the charged particles will move toward either the anode or the cathode, depending on the sign of their net charge. In addition to the particle movement (i.e., electrophoresis), the electric field also causes motion (i.e., electro-osmosis) of the dispersant/medium due to the negatively charged surface of the sample channel. The resulting flow has a parabolic velocity profile. By offsetting the total velocity profile, a net zero velocity flow (when summed over the depth of the sample channel) is created from which an electro-osmotic profile can be simulated. To eliminate the influence of the electro-osmotic flow, the PTA software (NTA 3.3) records the total apparent drift velocity (for each tracked particle) at different depths within the sample channel and subtracts the electro-osmosis component using the simulated profile. The resulting effective particle velocity (per unit electric field) are then converted into the corresponding electrophoretic mobility using the electric field strength. The zeta potential is then obtained on a particle-by-particle basis following Equation (1).

### 2.4. pH Measurements

Zeta potential is pH dependent and results should therefore be reported along with the pH values of the test suspension. In particular for the trueness assessment, the pH of all CRM samples was confirmed experimentally. The pH measurements were conducted potentiometrically at room temperature using a 744 pH Meter (Metrohm AG, Herisau, Switzerland) and a combined pH probe with integrated Pt1000 resistance temperature sensor (Solitrode 6.0228.000, Metrohm AG, Herisau, Switzerland). The glass bulb of the probe, with integrated double pin platinum electrode, was filled with 3 mol/L potassium chloride electrolyte solution. The linear measurement response was calibrated with two buffer CRMs at pH 4.00 and pH 9.00, and verified with another CRM at pH 7.00. All buffer solutions were purchased from Metrohm Belgium NV (Antwerp, Belgium).

### 2.5. Method Validation

According to the EURACHEM guideline [30], different method performance parameters need to be assessed during a validation study. The parameters that were evaluated during the ELS and PTA validation studies were robustness, limit of detection (LOD), limit of quantification (LOQ), working range, repeatability, intermediate precision and trueness. The performance parameter corresponding to the methods’ linearity (as known from traditional analytical methods such as liquid or gas chromatography) was not evaluated as the ELS and PTA methods are absolute methods whose signal responses do not require calibration with an electrophoretic mobility or zeta potential calibrant. Additionally, the parameter selectivity, which is the method’s capability to distinguish quantitatively between two or more particle populations, was not applicable as both methods were developed specifically for the analysis of monodisperse particle systems.

The validation studies were conducted by analyzing different materials (Table 1). Series of replicates performed under strict repeatability conditions were applied for assessing the working range and its associated lower and upper LOQ, as well as its lower LOD. Nested experimental designs were used for investigating the method’s precision (e.g., repeatability and intermediate), trueness and robustness. An overview of the use of the different materials throughout the different stages of the ELS and PTA validation studies is indicated in Table 3, where the (+) and (−) signs indicate whether a material was analyzed for investigating a given performance parameter, or not.

#### 2.5.1. Precision

The precision of a method represents the extent to which the different measurement results, obtained for a given material, agree. Three types of precision can be differentiated: repeatability, intermediate precision and reproducibility (between-laboratory precision) [30].

As the ELS and PTA methods are intended for single-laboratory use, the methods’ precision profiles were only characterized in terms of repeatability and intermediate precision.

For both validation studies, the different materials indicated in Table 3 were analyzed by two operators. The nested experimental designs involved distributing the number of replicates equally over different groups, which were different days, sample cells and temperature for ELS, and different days for PTA.

The use of a nested experimental design (in which only one parameter is varied at once) in combination with one-way analysis of variance (ANOVA) allows to separate the variances within and between groups from one another. The resulting mean squares within groups (*MSW*) and mean squares between groups (*MSB*) were used to calculate the relative standard deviations for method repeatability and intermediate precision, according to:

(4) $$RSD_{r}=100\cdot \frac{\sqrt{MSW}}{y_{m}}$$

(5) $$RSD_{\mathrm{ip}}=100\cdot \frac{\sqrt{\frac{MSB-MSW}{n_{r}}}}{y_{m}}$$

where, *RSD*_r_ and *RSD*_ip_ are the relative standard deviations for repeatability and intermediate precision, *n*_r_ is the number of measurement replicates per group, and *y*_m_ is the arithmetic average calculated from the different replicate results.

If *MSB* < *MSW*, then Equation (5) loses its validity due to the negative number under the square root. For these cases, an alternative approach was applied to calculate the relative variability between different groups, *RSD*_ip_^*^ [37]:

(6) $$RSD_{\mathrm{ip}}^{*}=100\cdot \frac{\sqrt{\frac{MSB-MSW}{n_{r}}+\frac{MSWe^{-\left(\frac{MSB}{MSW}\right)}}{n_{r}}}}{y_{m}}$$

The *RSD* values, which provide a quantitative expression of the repeatability and intermediate precision of the method, were considered reliable estimates for the relative standard uncertainties for repeatability, *u*_r_, and intermediate precision, *u*_ip_.

To increase the versatility of the ELS method, it was envisaged to validate the method for different types of measurement cells (see Table 2) and for different measurement temperatures in the range of 20 °C to 30 °C. Therefore, in addition to the classical setup in which replicates are spread over different days, additional precision studies were conducted to investigate the effect of the aforementioned method parameters. Rather than applying a multifactorial study design, the method parameters were examined using three independent one-factor-at-a-time studies. For each individual study, results for repeatability and intermediate precision were determined using Equations (4)–(6). The overall relative standard uncertainty for intermediate precision was then estimated by combining the worst-case intermediate precision uncertainties from the different studies:

(7) $$u_{\mathrm{ip}}=\sqrt{u_{\mathrm{ip}}^{2}\left(\mathrm{day}\right)+u_{\mathrm{ip}}^{2}\left(\mathrm{cell}\right)+u_{\mathrm{ip}}^{2}\left(\mathrm{temperature}\right)}$$

The method’s relative standard uncertainty for precision (*u*_prec_) was estimated following Equation (8), in which *u*_ip_ was combined with the worst-case *u*_r_, according to:

(8) $$u_{\mathrm{prec}}=\sqrt{\frac{u_{r}^{2}}{n_{r}}+\frac{u_{\mathrm{ip}}^{2}}{n_{d}}}$$

It must be noted that in Equation (8), the denominators *n*_r_ and *n*_d_, which correspond to the number of replicates and the number of measurement days, respectively, are set to 3 and 1. These values are not related to the number of replicates and days/groups of the conducted precision studies, but express the intended extent of the estimated uncertainty value, i.e., the estimated precision uncertainty is meant to be applicable to the average zeta potential calculated from three replicate results and obtained on a single measurement day. Such measurement scheme is common for routine testing.

The ELS experiments on DTS 1235, PS-A and ERM-FD306 were performed without applying any dilution to the materials.

For the PTA method, precision was assessed following a very similar approach, with that difference that the analysis of DTS 1235 (5 times diluted in Malvern Panalytical’s proprietary buffer) and ERM-FD305 (5000 times diluted in 10 mM borate buffer) was simply spread over different days. The relative standard uncertainties for *u*_r_, *u*_ip_ and *u*_prec_ were estimated according to the Equations (4)–(6) and (8).

#### 2.5.2. Trueness

Trueness refers to the closeness of agreement between a measurement result and a true value [30]. In other words, trueness reflects the level of ‘correctness’ of a measurement result. Therefore, trueness can only be determined by analyzing materials whose true values are known with an acceptable accuracy. Materials fulfilling those requirements are CRMs.

The trueness of ELS and PTA zeta potential results was quantitatively assessed in terms of experimental bias (Δ_bias_), which is the absolute difference between the certified value of a CRM (i.e., ERM-FD305 and ERM-FD306) and the average calculated from the replicate measurement results. Applying the procedure recommended by accredited CRM producers [38], the experimental bias is considered significant at a confidence level of 95% if Δ_bias_ > 2 × *u*_t_:

(9) $$u_{t}=\sqrt{u_{\mathrm{meas}}^{2}+u_{\mathrm{CRM}}^{2}}$$

where, *u*_t_ is the relative standard uncertainty for trueness, *u*_meas_ is the relative standard uncertainty associated to the mean of the zeta potential experimental results obtained for the CRM, and *u*_CRM_ is the relative standard uncertainty of the certified value. The latter is available from the CRM certificate.

The relative standard uncertainties of the experimental results obtained from the CRMs, *u*_meas_, were estimated using the same equation given in Equation (8), with the difference that both *n*_r_ and *n*_d_ now equals the effective number of replicates per day and the number of days over which the CRMs were analyzed.

#### 2.5.3. Working Range, LOD and LOQ

The working range of a method is the measurement range over which the method can be considered valid [30]. It is bracketed by the lower and upper limits of quantification (LLOQ and ULOQ). In chemical analysis, the LLOQ and ULOQ correspond to the lowest and highest analyte concentration that can be measured with an acceptable level of accuracy [30]. In analogy with the LLOQ and ULOQ, although not related to the working range in its strict meaning, one can also determine the lower and upper limits of detection as the lowest and highest analyte concentration that can be detected (but not reliably measured).

For chemical methods, the assessment of the working range and its limits of quantification can be relatively straightforward because of the clear relation between the analyte (i.e., a specific chemical substance) and the measurand (i.e., the quantity intended to be measured [39]). In fact, a relation between these fundamental concepts must, for obvious reasons always exist. However, for physical methods, such as the ELS and PTA methods presented in this work, the relationship is complicated as the measurability of the analyte (i.e., the particles) is strongly affected by a combination of factors that, in addition to a multitude of instrumental factors, are related to the particles’ optical properties, size and mass fraction. As the ELS and the PTA methods are intended primarily for polystyrene and silica particles whose diameters are neither too small nor too large, so as to not compromise the validity of Smoluchowski’s theory and the colloidal stability, a simplified approach based on dilution experiments could be applied for investigating the methods’ working ranges. The dilution experiments allow determining the mass fractions for which the signal-to-noise ratio becomes too low and for which multiple scattering and particle-particle interactions becomes significant. Multiple scattering occurs when the light scattered initially by a particle is scattered a second time by another particle.

The LLOQ and ULOQ of the ELS and PTA methods were assessed from dilution experiments performed on DTS 1235, PS-B, AWP4-B and ERM-FD305 (Table 3). To ensure that possible differences between results can be related to differences in mass fractions, all diluted test samples were analyzed under repeatability conditions, i.e., on one day and at a fixed temperature of 25 °C.

For ELS, single replicates were analyzed for both polystyrene materials; AWP-4 samples were analyzed in duplicate. For PTA, DTS 1235 and ERM-FD305 were analyzed in duplicate. ELS and PTA replicates (sample aliquot) were measured three times under repeatability conditions.

As ERM-FD305 is a CRM, the LLOQ and ULOQ were determined by comparing the measurement results with the certified value. In the case of the non-certified RMs, the LLOQ and ULOQ were determined statistically by means of the Grubbs’ outlier test at a confidence level of 95%.

The lower limit of detection (LLOD) of the ELS method depends on the amount of light scattered by the suspended particles that reaches the instrument’s photodetector. As the ELS instrument’s optical detection system is similar to that of dynamic light scattering (DLS), the minimum count rate condition for DLS was considered also valid for the ELS method, i.e., a suspension should generate a minimum count rate of 10 kilocounts per second (kcps) in excess of the intensity of the light scattered by the particle-free dispersant [40].

#### 2.5.4. Robustness

Robustness is a measure for the method’s capacity to obtain similar results when perturbed by small variations in procedural or method parameters [30]. A method is considered ‘sufficiently robust’ when the uncertainty of the measurement result is not significantly increased by the imposed variation.

For the ELS method, the robustness was assessed for the different cells and temperatures using the data obtained from the precision studies (Section 2.5.1). It must be noted that in case the method was found not robust with respect to a certain parameter level, the results corresponding to the given parameter level were excluded from the precision study.

In contrast to the ELS validation study, no specific method parameters were varied in a systematic manner during the PTA validation study. For PTA, the acquisition and the analysis of the videos require the operator to define optimal values for the focus, camera level and the detection threshold. The camera level, which is used to distinguish between light scattered by the particles and stray light from other sources, is set prior to video acquisition. The detection threshold, which is set before video processing, determines the minimum gray scale value of any particle to be recognized as a particle. As the exact settings depend on the quality of the test samples, it was decided not to organize a dedicated robustness study for the abovementioned parameters, but to allow the parameters to vary during the experiments conducted for assessing method precision.

By having both the ELS and PTA measurements performed by two different operators, the potential variation due to sample preparation and different operators is automatically included in the uncertainty contribution for precision.

The robustness was confirmed statistically using the *F*-statistic in one-way ANOVA, the Student’s *t*-test and the Kruskal-Wallis *H* test, all at a confidence level of 95%. The Kruskal-Wallis *H* test, which is a nonparametric robust method that compares the medians of different groups, was used when the null hypothesis for group means could not be tested due to unequal variances. Unequal variances violate the validity of one-way ANOVA. Data normality was verified graphically using normal probability plots (see Supplementary Materials).

#### 2.5.5. Measurement Uncertainty

The expanded relative measurement uncertainties (*U*) for zeta potential results obtained with the validated methods were estimated by combining the relative standard uncertainties of precision and trueness using the root-mean-square manner [31]. The values are valid for the average of triplicate zeta potential results all obtained under repeatability conditions (i.e., on one day). A coverage factor, *k* = 2, was used to express the uncertainties on an approximate 95% confidence interval.

(10) $$U=k\cdot \sqrt{u_{\mathrm{prec}}^{2}+u_{t}^{2}}$$

## 3. Results and Discussion

### 3.1. Precision

**Figure 1:**
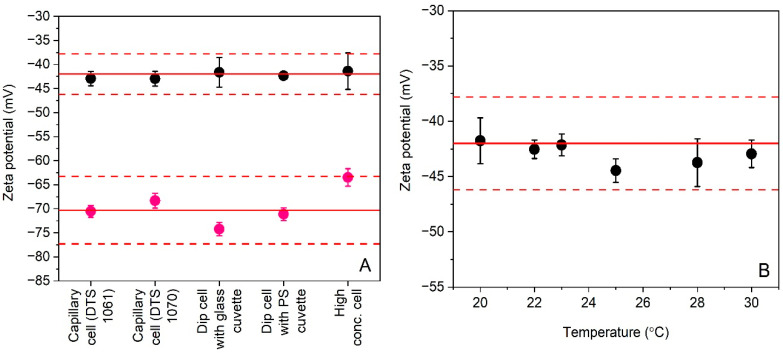
Average zeta potential results (*n*_r_ = 4) for polystyrene particles DTS 1235 (black circles) and PS-A (purple circles) obtained by ELS during precision studies when using different types of measurement cells (**A**) and different measurement temperatures (**B**). Error bars correspond to standard deviations. Solid and dashed lines reflect the zeta potential reference values and uncertainties assigned to the materials by the manufacturers.

Measurement uncertainties estimated from validation data are often reduced by keeping fixed as many method parameters as possible. This approach is perfectly justifiable when a laboratory needs to analyze a specific type of sample on a routine basis. However, the main risk of such narrow scope is that estimated measurement uncertainties may no longer be representative, and thus useful, when one of the crucial method parameters is changed, for instance, as a response to a changing sample quality. While the ELS method has been validated particularly to support the development and production of colloidal silica CRMs by EC-JRC, it was decided that the method should also be applicable to polystyrene particles, which are popular materials for verification and calibration of different particle size analysis methods. Allowing some of the ELS method’s crucial parameters (i.e., type of measurement cell and temperature) to vary within defined boundaries increases significantly the versatility of the validated method. Zeta potential results obtained by ELS for different measurement cells and temperatures are shown in Figure 1.

Prior to the assessment of the method’s precision, the datasets shown in Figure 1 were subjected to statistical scrutiny to evaluate the method’s robustness. The statistical outcome, which is discussed in detail in Section 3.3, concluded that the ELS method is only robust for the two capillary cells (DTS 1061 and DTS 1070) and for the dip cell when used in combination with a standard polystyrene cuvette. In addition, the ELS method was found sufficiently robust over the temperature range of 20 °C to 30 °C.

Only the datasets obtained with the robust ELS method were used for estimating the relative standard uncertainties for precision.

An overview of the relative standard uncertainties for repeatability and intermediate precision of the ELS and PTA methods are shown in Table 4 and Table 5, respectively. The values for *n*_r_ and *n*_ip_ correspond to the number of measurement replicates within a group and the number of different groups (or the number of levels varied for a given method parameter).

For the tested materials and method parameters, it can be concluded that the repeatability of the ELS method remains relatively constant with uncertainties in the range of 2.0% to 2.5%, independent of whether measurements are performed in duplicate or quadruplicate. When varying the temperature within the chosen temperature range, the uncertainty increases to 3.5%. Combining the intermediate precision uncertainties reflecting the between-day, between-cell and between-temperature variability gives a combined relative standard uncertainty for intermediate precision of 3.4%. The latter was then combined with the worst-case uncertainty for repeatability (i.e., 3.5%) giving a combined relative standard uncertainty for method precision of 3.6%.

The repeatability of the PTA method is comparable to the repeatability of the ELS method. However, the performance of the method was more repeatable when analyzing ERM-FD305 than for DTS 1235. The between-day variability of the method was not significantly affected by the test material. Combining the worst-case uncertainties for repeatability and intermediate precision gives a relative standard uncertainty for precision of 2.4%.

For both the ELS and the PTA methods, the combined relative standard uncertainties for precision are valid for triplicate zeta potential results obtained from measurements performed under repeatability conditions.

### 3.2. Trueness

The trueness of the zeta potential results obtained by the ELS and PTA methods was evaluated by analyzing the colloidal silica certified reference materials ERM-FD305 and ERM-FD306. The significance of the experimental bias (Δ_bias_), calculated from the certified zeta potential values, *u*_CRM_, and the mean zeta potential of the experimental results, *u*_meas_, was assessed against its associated (absolute) standard measurement uncertainty, *u*_t_. The results of the trueness evaluation and of the comparative analysis of the CRMs are summarized in Table 6.

As can be seen, the experimental biases were always smaller than 2 × Δ_bias_, thus indicating that the ELS and PTA methods were able to accurately measure the zeta potential of the analyzed CRMs. The significant difference in magnitude between *u*_meas_ and *u*_CRM_ is not unusual, as the uncertainty of a certified value includes important and significant uncertainty contributions from, at least, batch homogeneity, stability and characterization. For ERM-FD306, which was analyzed in duplicate over five days, *u*_meas_ was estimated using an equation similar to Equation (8), with that difference that *n*_r_ and *n*_d_ were equal to 2 and 5, respectively. Instead, *u*_meas_ for the results obtained on ERM-FD305 was estimated as the standard error of the replicate results.

### 3.3. Working Range, LOD and LOQ

Zeta potential data obtained for the polystyrene materials PS-B and DTS 1235 on up to 16 and 8 different particle mass fractions, respectively, as well as for colloidal silica AWP4-B on up to 11 mass fractions and for three different cell types, have been investigated by the Grubbs’ outlier test for statistically significant (α = 0.05) differences among different mass fractions. Within each set of experiments, all method parameters remained constant to ensure that potential differences could be primarily attributable to changes in the method’s signal-to-noise ratios as a response to changes in particle mass fraction.

**Figure 2:**
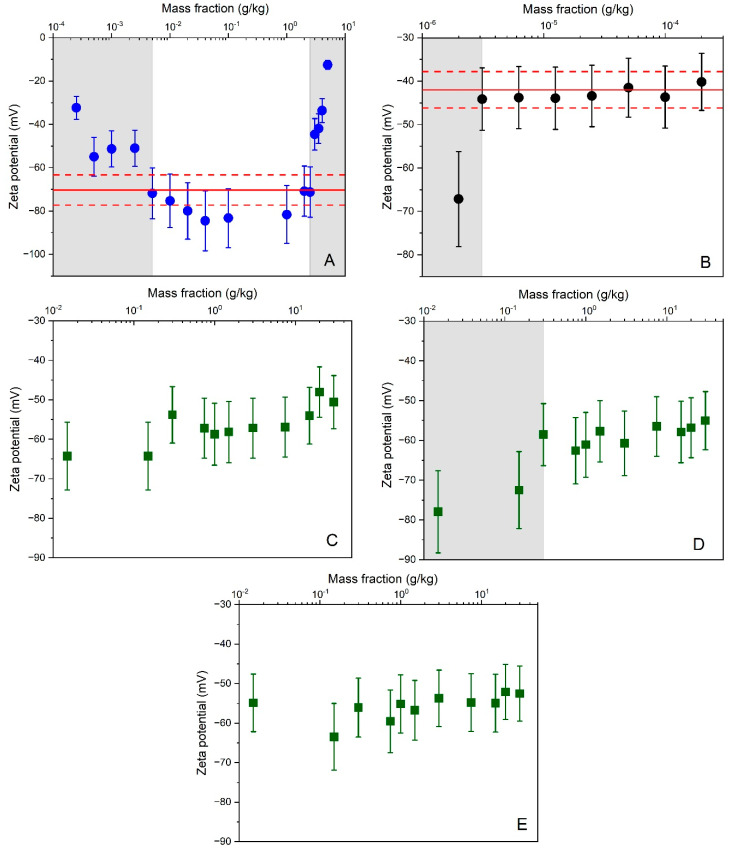
Average zeta potential results as a function of particle mass fractions obtained by ELS for different materials and cells: (**A**) PS-B + capillary cell DTS 1061 with 4 mm OP; (**B**) DTS 1235 + capillary cell DTS 1061 with 4 mm OP; (**C**) AWP4-B + capillary cell DTS 1070 with 4 mm OP; (**D**) AWP4-B + dip cell with PS cuvette with 10 mm OP; (**E**) AWP4-B + high concentration cell with 2 mm OP. Error bars correspond to expanded (*k* = 2) measurement uncertainties. Solid and dashed lines reflect the zeta potential reference values and uncertainties assigned to the materials by the manufacturers. Results located in gray shaded areas were identified as outside the working range.

For PS-B (Figure 2A), zeta potential results obtained for test samples with mass fractions outside the range between 5 mg/kg and 2.5 g/kg were flagged as statistical outliers. Within this range, the ELS method yielded stable zeta potential results that, at least, partly overlapped with the indicative reference range assigned by the manufacturer. Consistent results were also obtained across the different test samples prepared from DTS 1235 (Figure 2B). Only for the sample with lowest mass fraction (i.e., dilution factor of 100), significantly lower zeta potential values were obtained. When comparing the two working ranges, one can note a clear shift towards lower mass fractions for DTS 1235. Indeed, although both materials consist of plain polystyrene particles dispersed in an aqueous medium, DTS 1235 can be measured at a 2500 times lower particle mass fraction than PS-B. This observation can be readily explained by the materials’ difference in particle size. It is well known that for particles smaller than the wavelength of the illuminating light, the intensity of scattered light increases proportionally with the particle diameter to the sixth power [41]. The particles of DTS 1235 are twice the size of the PB-S particles and thus by virtue scatter the light much more intensely than the smaller particles of PB-S. These results confirm that the working range, and its limits, are particle size dependent.

To investigate the working range of the ELS method for colloidal silica, 11 different dilutions were prepared in 10 mM borate buffer from the highly concentrated material AWP4-B. As can be seen from the results depicted in Figure 2C–E, similar zeta potential results were generally obtained across the different samples and for the three different types of cells (i.e., capillary cell DTS 1070, dip cell with standard PS cuvette, high concentration cell). These results are remarkable, as the ULOQ was expected to decrease with increasing optical path length. Instead, it was found that the LLOQ increased from 0.015 g/kg when using cells with an optical path length of 2 mm and 4 mm (Figure 2C,E), to 0.3 g/kg when using a cell with a standard optical path length of 10 mm (Figure 2D). While the influence of sample volume was not in the focus of our study, it is worth mentioning that the small path length of the high concentration cell is matched with a low volume electrode chamber that allows the examination of sample volumes as low as 150 μL. Hence, the high concentration cell may also be useful for precious samples or samples with limited volumes.

The determination of the limit of detection (LOD) can be a challenging exercise as it is not always straightforward to demonstrate that a measurement signal is no longer related to the target measurand. Sometimes, the LLOD is estimated as three times the standard deviation from results by repeated measurements on a blank sample [30]. When measuring physical properties of particles, this approach is not suitable since the measurement signal from particle-free samples cannot be meaningfully related to a particle-related measurement performance. For the ELS method, the derived count rate, or the number of photons per second that reached the detector, was used as an indicator to predict whether the measurement signal was mainly generated by the particles and not by the dispersant. The derived count rates obtained from the different particle-free buffers were in the range of 10–30 kcps. For all tested samples, a derived count rate of >50 kcps was found, suggesting that the particle mass fractions were above the LLOD.

**Figure 3:**
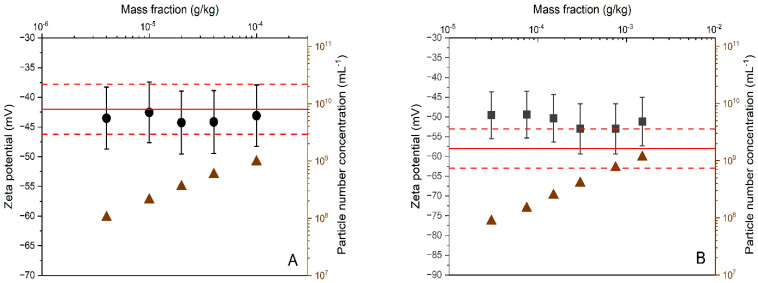
Average zeta potential and particle number concentration (triangles) results as a function of particle mass fractions obtained by PTA for DTS 1235 (**A**) and ERM-FD305 (**B**). Error bars correspond to expanded (*k* = 2) measurement uncertainties. Solid and dashed lines reflect the zeta potential reference values and uncertainties assigned to the materials by the manufacturers.

The working range of the PTA method was evaluated using the same methodology applied for the ELS validation study. For the dilutions prepared from DTS 1235, all calculated zeta potential results overlapped with the −42 mV line that represents the reference value assigned by the manufacturer (Figure 3A). In addition, no systematic trends or inconsistencies were detected from statistical scrutiny. Consistency was also demonstrated among the zeta potential results obtained for the certified reference material, ERM-FD305 (Figure 3B). However, in contrast to DTS 1235, the zeta potential results obtained on ERM-FD305 are systematically above the reference line and overlap with the certified range of −37.8 mV to −46.2 mV is only achieved by the results’ confidence intervals (i.e., error bars), which correspond to an expended measurement uncertainty of 12% (see further). Despite this visual trend, the zeta potential results obtained for the different dilutions are not significantly different from the certified range confirming the mass fraction working range of the method.

Approaching the limits of the working range can lead to a loss of method repeatability. Such deterioration can be monitored from the standard deviations calculated from replicate results. For the ELS methods, no meaningful standard deviations could be calculated from the two replicate results. For the PTA method, measurements were also conducted in duplicate and each replicate was three times measured under repeatability conditions. As the sample suspension in the measurement volume was replaced by a fresh amount of suspension prior to each new measurement repeat, the six individual results can be considered as independent results, thus giving sufficient degrees of freedom for calculating a reliable and meaningful standard deviation. The relative standard deviations of the PTA results obtained for DTS1235 and ERM-FD305 range from 1.3% to 2.6% and from 0.1% to 1.3%, respectively. The variation of the standard deviations was neither systematic nor correlated to the mass fraction.

For PTA, the lower and upper limits of the working ranges can be regarded as the LLOQ and ULOQ. For silica, the LLOQ and ULOQ are 0.02 mg/kg and 0.1 g/kg, respectively. For polystyrene, the limit values are 0.15 mg/kg and 1.5 g/kg. No conclusions on the LLOD can be drawn from the experimental data. The measured particle number concentration along with the minimum threshold of 10^7^ particles/mL (recommended by the manufacturer) may be used as indicator to decide whether zeta potential results are close are to the LLOD. For the ELS method, the LLOQ and ULOQ of the mass fraction working ranges are 0.015 g/kg and 30 g/kg (for silica) and 0.003 mg/kg and 2.5 g/kg (for polystyrene).

### 3.4. Robustness

For the ELS method, the parameters examined were the type of the measurement cell (and its inherently linked sample volume and electrode material) and the test temperature. The ability of using different cells provides additional versatility to the method as sample volumes as small as 0.2 mL can be analyzed. The effect of sample temperature is assumed less critical as the measured temperature and the dynamic viscosity value, required as input quantity in Equation (1), are coupled. The assessment of robustness was based on the experimental data obtained in the precision study (Figure 1).

Figure 1A shows that all zeta potential results fall within the 10% confidence intervals around the two reference values, but the data for DTS 1235 are more consistent than the data for PS-A. The average values were calculated from only four replicate results. However, the normal probability plots (Figure S1A,B) do not suggest that the data of the different groups are not normally distributed. In addition to the excellent agreement for DTS 1235, one can discern a substantial difference in size between the error bars for data obtained with the high concentration cell and the dip cell with glass cuvette, and those associated to data obtained with the other cells. This phenomenon was not observed for PS-A. Due to the unequal variances of the groups, the null hypothesis could not be tested with the *F*-statistic from one-way ANOVA. As an alternative, the Kruskal-Wallis *H* test was used to compare the group medians. At a confidence level of 95%, the median values of the different cell type groups were not significantly different from one another. Nevertheless, the unequal variances are important indicators that, at least for DTS 1235, the ELS method performed differently depending on the type of cell used. Because the variances of the data obtained with the previous and the new model of the folded capillary cells (DTS 1061 and DTS 1070) were similar in size, a Student’s *t*-test was applied to the two datasets. At a confidence level of 95%, it was found that both datasets were not significantly different (*p* > 0.05). It is also noted that the difference in variance between the folded capillary cells and the PS dip cell is significant, but this is due to the exceptionally small variance of the results of the PS dip cell. As there was no evidence that the data of PS-A are not normally distributed (Figure S1B) and because the data of the different groups exhibit similar variances, one-way ANOVA was employed to compare the different group means. Based on the *F*-statistic and its associated probability value, the null hypothesis was rejected on a 95% confidence level. Paired *t*-tests demonstrated that the zeta potential results obtained with the high concentration cell and with the glass dip cell were significantly different from the zeta potential results obtained with the folded capillary cells and the PS dip cell.

The robustness of the ELS method for different temperatures is illustrated graphically in Figure 1B. A visual inspection of the error bars indicates significantly larger variances for the data at 20 °C and 30 °C. According to the Kruskal-Wallis *H* test, the different group medians were not significantly different at a confidence level of 95%. The unequal variances, in particular those of the datasets corresponding to the lower and upper temperatures of the tested range, may indicate that the ELS method is less robust. However, each deviating variance was triggered by a single statistical outlier. According to one-way ANOVA, which was performed on the datasets exhibiting equal variances, the null hypothesis should be rejected based on the *F*-statistic and its probability (*p* = 0.04). While significant differences amongst groups were demonstrated statistically, all results agreed with the assigned reference value and its associated expanded uncertainty. Therefore, it can be reliably assumed that the ELS method is sufficiently robust for zeta potential measurements performed within the tested temperature range of 20 °C to 30 °C.

The PTA method offers less versatility than the ELS method, as it uses a fixed sample cell assembly. Instead, the performance of the PTA method can be more sensitive to operator bias as for each sample both the focus, camera level and the detection threshold need to be adjusted. For repeated measurements on a homogeneous sample, these settings may be either fixed or set automatically by the instrument software. The three parameters were not investigated systematically. Instead, they were set either automatically by the instrument software or manually during the experiments that were conducted for assessing the precision of the method. For ERM-FD305 (5000 times diluted), the camera level and detection threshold were set in the ranges of 10 to 15 and 6 to 12, respectively. For DTS 1235 (5 times diluted), the camera level and detection threshold ranged from 3 to 7 and from 7 to 21.

### 3.5. Measurement Uncertainty

**Figure 4:**
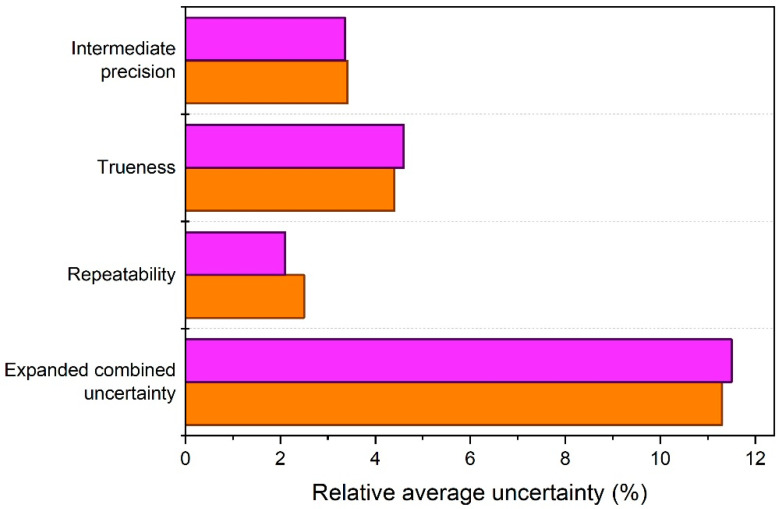
Relative average standard uncertainties and the relative expanded combined uncertainty estimated for triplicate zeta potential results from the validated ELS (orange) and PTA (purple) methods.

The relative expanded (combined) uncertainties, *U*, were estimated by combining the relative standard uncertainties from precision and trueness, and by applying a coverage factor, *k* = 2 (Equation (10)). The uncertainty budgets established for the ELS and PTA validated methods are depicted graphically in Figure 4. The uncertainties are averages, which account for triplicate zeta potential results obtained on one day. The relative expanded uncertainties for triplicate zeta potential results from ELS and PTA are 11.3% and 11.5%, respectively, or 12% when rounded up.

As can be seen from the bar chart, the uncertainty values of the two methods are very similar, not only with respect to the final expanded combined uncertainties, but also with respect to their individual standard uncertainties. Sometimes, the combined uncertainty can be heavily influenced by one dominant component. However, for both the ELS and PTA methods, the contributions from the three uncertainty sources, which have been extensively discussed above, are all of comparable magnitudes. PTA does offer a slightly better repeatability, but this is not reflected in expanded uncertainty. The latter shows that increasing the number of measurement replicates will only have an insignificant effect on the combined uncertainties. The relative standard uncertainties for intermediate precision are nearly identical, despite the different approaches applied during the precision studies of the two methods. For the ELS method employed with a given type of measurement cell and at a given temperature, the uncertainty for intermediate precision reflecting only day-to-day variation and operator uncertainties is about 2.1%. When comparing this value with intermediate precision uncertainty from PTA (i.e., 3.4%), which also reflects day-to-day variation and operator uncertainties, it can be concluded that zeta potential results from PTA are more sensitive to operator bias, although no significant between-operator differences were detected from the precision data.

### 3.6. Method Transfer

The ELS and PTA measurement procedures were developed and validated specifically for polystyrene and silica particles, as these particles are often used to serve as RM for nanoparticle size analysis [42]. Furthermore, so-called full validation studies were applied to allow possible transfer to other laboratories equipped with the same instruments. As described by Varenne and colleagues, method transfer is only successful when the receiving laboratory is able to get comparable results [43].

If laboratories measure similar sample systems and operate the methods within their validated scopes, then the methods can be readily implemented and receiving laboratories only need to verify the accuracy of the results. Alteration of method parameters can significantly affect the method performance characteristics. In that case, laboratories should carefully revalidate the measurement procedures for their own applications. The degree of revalidation required depends on the nature of the changes. For instance, recent research has pointed at the importance of the choice of electrode material when measuring the electrophoretic mobility of proteins such as bovine serum albumin [44]. The researcher concluded that at ionic strengths of 10 mM and higher, PS dip cells combined with Pt and Pd electrodes yield lower electrophoretic mobility results than platinized Pt electrodes. Other researchers found that for chitosan, a heteroglycan which has gained attention as a potential drug delivery system in cancer therapy, an increase in ionic strength results in a decrease of electrophoretic mobility [45,46]. Liao et al. and Novak et al. demonstrated that particle morphology, type of buffer, and surfactant concentration are important factors when measuring the zeta potential of titanium dioxide [47,48].

For laboratories in need of analyzing particles with positive surface charges (e.g., numerous nanocomposites or metal oxides at low pH), we recommend the reader to consult the ELS procedure that was validated by Varenne et al. [26].

## 4. Conclusions

In this study, we developed and validated two optical methods, ELS and PTA, for measurement of zeta potential of silica and polystyrene particles. We comprehensively evaluated the key method performance characteristics by analyzing a suit of fit-for-purpose reference materials, including two recently produced colloidal silica CRMs. The successful validation, which includes robustness, working ranges, limits of quantification, precision and trueness, provided satisfactory accuracy with an expanded uncertainty of 12%.

The methods were developed and validated primarily to support the production of future colloidal silica CRMs by EC-JRC. However, since we have demonstrated the absence of systematic experimental biases, the validated methods can also be transferred with a minimum of effort to other laboratories, provided they analyze similar sample systems. For those laboratories that cannot implement the methods directly, our contribution provides practical guidance on how to conduct validation studies and estimate measurement uncertainties.

Future work could focus on extending the scope of the methods for other types of particles, such as particles with a net positive surface charge and/or particles of other elemental composition.

## Figures and Tables

**Table 1:** Colloidal polystyrene and silica reference and test materials used during method validation, and their relevant properties.

Material	Type	Dispersant	Nominal Particle Diameter (nm)	Reference Zeta Potential (mV) ^1^	Mass Fraction (g/kg)	Metrological Status
DTS 1235	PS	NDA, pH 9	300	−42.0 ± 4.2	2 × 10^−4^	RM
PS-A	PS	10 mM NaCl	150	−70.3 ± 7.0	0.01	RM
PS-B	PS	10 mM NaCl	150	−70.3 ± 7.0	10	RM
AWP4-A	SiO_2_	10 mM borate, pH 9	140	N/A	15	Test sample
AWP4-B	SiO_2_	10 mM borate, pH 9	140	N/A	30	Test sample
ERM-FD305 [32]	SiO_2_	10 mM borate, pH 9	140	−58 ± 5	1.5	CRM
ERM-FD306 [33]	SiO_2_	10 mM borate, pH 9	140	−56 ± 4	22	CRM

^1^ Uncertainty value corresponds to a confidence level of approximately 95%; N/A: not available; NDA: identification and composition of buffer is subject to a non-disclosure agreement between Malvern Panalytical and the JRC; PS: polystyrene.

**Table 2:** Relevant parameters and parameter levels of the ELS measurement procedure applied during the precision and trueness experiments of the validation study.

Method Parameters	Parameter Levels	
	ELS	PTA
Dispersant viscosity	0.889 mPa s (water) [35] ^1^	
Dispersant relative permittivity	80 (water) [36]	
Temperature	(20–30) °C	(25.0 ± 0.1) °C
Particle refractive index	1.59 (polystyrene) [36] 1.46 (silica) [36]	
Theoretical model, *f*(*κa*)	Smoluchowski	
Type of measurement cell	Folded capillary cell (DTS 1061, 0.75 mL, 4 mm OP, polycarbonate, gold plated beryllium copper alloy electrodes Folded capillary cell (DTS 1070, 0.75 mL, 4 mm OP, polycarbonate, gold plated beryllium copper alloy electrodes) ^2^ Dip cell (ZEN 1002, 1.5 mL, 10 mm OP, PEEK palladium electrodes submerged in suspension contained in standard polystyrene cuvette,)	Gasket (NTA4136) of 0.58 mm thickness sandwiched between the optical flat and glass top plate; platinum electrodes
	High concentration/low volume cell (ZEN 1010, 0.2 mL, 2 mm OP, quartz cell)	
Light source	He-Ne laser 633 nm	Laser diode 405 nm
Detector type	Avalanche photodetector	Orca-Flash 2.8 scientific CMOS
Collection angle	13°	N/A
Voltage selection	Automatic	24 V
Attenuator selection	Automatic	N/A
Analysis mode	Automatic	N/A
Measurement time	120 s	120 s
Delay between measurements	0 s	10 s
Focus	N/A	Automatic and manual
Camera level	N/A	Automatic and manual
Detection threshold	N/A	Automatic and manual

^1^ The viscosity was automatically re-calculated by the instrument software for the measured temperature; OP, optical path; ^2^ The folded capillary cell DTS 1070 has been developed and introduced by Malvern Panalytical to improve the reproducibility of zeta potential results. It nowadays replaces the previous model, DTS 1061; N/A: not applicable.

**Table 3:** Use and role of different particulate materials throughout the ELS and PTA validation studies.

Material	ELS/PTA			
	Precision	Trueness	Working Range, LOD, LOQ	Robustness
DTS 1235	+/+	−/−	+/+	+/+
PS-A	+/−	−/−	−/−	+/−
PS-B	−/−	−/−	+/−	−/−
AWP4-A	−/−	−/−	−/−	−/−
AWP4-B	−/−	−/−	+/−	−/−
ERM-FD305	−/+	+/+	−/+	−/+
ERM-FD306	+/−	+/−	−/-	−/−

**Table 4:** Repeatability and intermediate precision results of the ELS method.

Material	Zeta Potential (mV)	*n*_r_	*n*_ip_	*u*_r_ (%)	*u*_ip_ (%)
-	Between-days (fixed: T = 25 °C, cell = DTS 1061)				
DTS 1235	−43.1	2	5	2.5	2.1
ERM-FD306	−53.9	2	5	2.3	0.8 ^1^
-	Between-cells (fixed: T = 25 °C, day = 1)				
DTS 1235	−42.7	4	5	2.0	0.4 ^1^
PS-A	−70.0	4	5	2.0	2.2
	Between-temperatures (fixed: cell = DTS 1061, day = 1)				
DTS 1235	−42.9	4	6	3.5	1.6

^1^ Calculated as *RSD*_ip_^*^.

**Table 5:** Repeatability and intermediate precision results of the PTA method.

Material	Zeta Potential (mV)	*n*_r_	*n*_ip_	*u*_r_ (%)	*u*_ip_ (%)
-	Between-days (variable camera level and detection threshold)				
DTS 1235	−44.1	3	6	2.1	3.0
ERM-FD305	−52.4	3	6	1.0	3.4

**Table 6:** Results of the trueness assessments.

CRM	Zeta Potential (mV)	*n*_r_	*n*_d_	pH	Δ_bias_ (mV)	*u*_meas_ (mV)	*u*_CRM_ (mV)	*u*_t_ (mV)	*u*_t_ (%)	Significant Bias ^1^
-	ELS method									
ERM-FD305	−55.8	6	3	9.0	2.2	0.5	2.5	2.5	4.4	No
ERM-FD306	−53.9	10	5	9.0	2.1	0.6	2.0	2.1	3.8	No
-	PTA method									
ERM-FD305	−54.3	9	3	8.9	3.7	0.9	2.5	2.7	4.6	No

^1^ Confidence level of approximately 95%.

## Data Availability

The data presented in this study are available on request from the corresponding author.

## Supplementary Materials

The following are available online at https://www.mdpi.com/1996-1944/14/2/290/s1, Figure S1: Normal probability plots for zeta potential obtained by ELS for (A) DTS 1235 and different cells, (B) PS-A and different cells and (C) DTS 1235 and different temperatures.

## Abbreviations

ANOVA	analysis of variance
*a*	radius of a sphere
CRM	certified reference material
DLS	dynamic light scattering
*E*	electric field strength
ELS	electrophoretic light scattering
ISO	International Organization for Standardization
*k*	coverage factor
kcps	kilocounts per second
LLOD	lower limit of detection
LLOQ	lower limit of quantification
*MSB*	mean squares between groups
*MSW*	mean squares within groups
*n*	refractive index of medium
*n*_d_	number of measurement days
*n*_ip_	number of groups in an intermediate precision study
*n*_r_	number of replicates within groups
OP	optical path (length)
PALS	phase analysis light scattering
PS	polystyrene
PTA	particle tracking analysis
REACH	registration, evaluation, authorisation and restriction of chemicals
RM	reference material
*RSD*_r_	relative standard deviation for repeatability
*RSD*_ip_	relative standard deviation for intermediate precision
*RSD*_ip_^*^	relative standard deviation for intermediate precision (if *MSB* < *MSW*)
*U*	Relative expanded uncertainty
ULOQ	upper limit of quantification
*u*_CRM_	uncertainty of certified value
*u*_ip_	intermediate precision uncertainty
*u*_meas_	measurement uncertainty used in trueness assessment
*u*_prec_	precision uncertainty
*u*_r_	repeatability uncertainty
*u*_t_	trueness uncertainty
V	electrophoretic velocity
*y*_m_	arithmetic average calculated from replicate results
Δ_bias_	experimental bias
Δ*w*	Doppler frequency shift
*ε*	permittivity of the medium
*ζ*	zeta potential
*η*_0_	dynamic viscosity of the medium
*θ*	angle between the incident light and scattered light
*κ*	reciprocal of the Debye double layer thickness
*λ*_0_	wavelength of laser light (in vacuum)
*μ*	mean electrophoretic mobility
*ξ*	angle between the scattered light and the orientation of the electric field

## Appendix A

The ELS methodology implemented by Malvern Panalytical is based on a patented technology referred to as M3-PALS (mixed mode measurement—phase analysis light scattering). The M3 method performs two consecutive measurement sequences for each *ζ*-potential measurement. During the first sequence, the applied electric field is reversed slowly (once per second) while during the second sequence the electric field is reversed rapidly. Both the slow field reversal (SFR) and fast field reversal (FFR) portions are presented in a typical zeta potential plot. The SFR method is applied to allow the suspension flow to stabilize and to reduce the polarization of the cell electrodes which is inevitable in a conductive solution. In FFR, low voltages are rapidly oscillated for about 22 times in order to allow the particles to reach their terminal velocities. To minimize the effect of electro-osmosis, the particle velocities are measured at the center of the cell. The magnitude of the oscillations, or the nominal voltage applied via the drive circuit to the cell electrodes, is automatically set by the instrument software depending on the selected measurement cell and on the *f*(*κa*) selection. The voltage, which is nominally applied to the electrodes of the measurement cell, will deviate from the actual or effective voltage experienced by the sample in the cell. The effective voltage is calculated by the software by measuring the conductivity and the current. The PALS technology is used to measure the phase shift between the incident/reference light and the light scattered by the particles, rather than measuring directly the frequency shift. Detection of a phase change is more sensitive to changes in mobility, than detection of a frequency shift. The phase shift is used to accurately determine the frequency shift. The combination of M3 and PALS allows measurements of high conductivity samples and samples of particle with characterized by a low electrophoretic mobility.

## References

[1] Danish Ecological Council and Danish Consumer Council The Nanodatabase. https://nanodb.dk/.

[2] Foss Hansen S., Heggelund L.R., Revilla Besora P., Mackevica A., Boldrin A., Baun A. (2016). Nanoproducts—What is actually available to European consumers?. Environ. Sci. Nano.

[3] Noorlander C.W., Kooi M.W., Oomen A.G., Park M.V.D.Z., Vandebriel R.J., Geertsma R.E. (2015). Horizon scan of nanomedicinal products. Nanomedicine.

[4] Oberdörster G., Stone V., Donaldson K. (2007). Toxicology of nanoparticles: A historical perspective. Nanotoxicology.

[5] Gupta R., Xie H. (2018). Nanoparticles in daily life: Applications, toxicology and regulations. J. Environ. Pathol. Toxicol. Oncol..

[6] European Commission (2006). Regulation (EC) No 1907/2006 of the European Parliament and of the Council of 18 December 2006 concerning the Registration, Evaluation, Authorisation and Restriction of Chemicals (REACH), establishing a European Chemicals Agency, amending Directive 1999/45/EC and repealing Council Regulation (EEC) No 793/93 and Commission Regulation (EC) No 1488/94 as well as Council Directive 76/769/EEC and Commission Directives 91/155/EEC, 93/67/EEC, 93/105/EC and 2000/21/EC. Off. J. Eur. Union.

[7] European Commission (2018). Commission Regulation (EU) 2018/1881 of 3 December 2018 amending Regulation (EC) No 1907/2006 of the European Parliament and of the Council on the Registration, Evaluation, Authorisation and Restriction of Chemicals (REACH) as regards Annexes I, III, VI, VII, VIII, IX, X, XI, and XII to address nanoforms of substances. Off. J. Eur. Union.

[8] European Commission (2020). Commission Regulation (EU) 2020/878 of 18 June 2020 amending Annex II to Regulation (EC) No 1907/2006 of the European Parliament and of the Council concerning the Registration, Evaluation, Authorisation and Restriction of Chemicals (REACH). Off. J. Eur. Union.

[9] European Commission (2011). Commission Recommendation (EU) 2011/696 of 18 October 2011 on the definition of nanomaterial. Off. J. Eur. Union.

[10] Braun A., Couteau O., Franks K., Kestens V., Lamberty A., Linsinger T., Roebben G. (2011). Validation of dynamic light scattering and centrifugal liquid sedimentation methods for nanoparticle characterisation. Adv. Powder Technol..

[11] Hole P., Sillence K., Hannell C., Maguire C.M., Roesslein M., Suarez G., Capracotta S., Magdolenova Z., Horev-Azaria L., Dybowska A. (2013). Interlaboratory comparison of size measurements on nanoparticles using nanoparticle tracking analysis (NTA). J. Nanopart. Res..

[12] De Temmerman P.-J., Lammertyn J., De Ketelaere B., Kestens V., Roebben G., Verleysen E., Mast J. (2014). Measurement uncertainties of size, shape and surface measurements using transmission electron microscopy of near-monodisperse, near-spherical nanoparticles. J. Nanopart. Res..

[13] Varenne F., Botton J., Merlet C., Beck-Broichsitter M., Legrand F.-X., Vaughtier C. (2015). Standardization and validation of a protocol of size measurements by dynamic light scattering for monodispersed stable nanomaterial characterization. Colloids Surf. A..

[14] Kestens V., Bozatzidis V., Ramaye Y., Roebben G. (2017). Validation of a particle tracking analysis method for the size determination of nano- and microparticles. J. Nanopart. Res..

[15] Langevin D., Raspaud E., Mariot S., Knyazev A., Stocco A., Salonen A., Luch A., Haase A., Trouiller B., Relier C. (2018). Towards reproducible measurement of nanoparticle size using dynamic light scattering: Important controls and considerations. NanoImpact.

[16] Antúnez Domínguez J.M., Ramaye Y., Dabrio M., Kestens V. (2020). Validation of a homogeneous incremental centrifugal liquid sedimentation method for size analysis of silica (nano)particles. Materials.

[17] Thompson M., Ellison S.L.R., Wood R. (2002). Harmonized guidelines for single-laboratory validation of methods of analysis. Pure Appl. Chem..

[18] ECHA (2019). Guidance on Information Requirements and Chemical Safety Assessment—Appendix R.6-1 for Nanoforms Applicable to the Guidance QSARs and Grouping of Chemicals.

[19] Stone V., Gottardo S., Bleeker E.A.J., Braakhuis H., Dekkers S., Fernandes T., Haase A., Hunt N., Hristozov D., Jantunen P. (2020). A framework for grouping and read-across of nanomaterials—Supporting innovation and risk assessment. Nano Today.

[20] Varsou D.-D., Afantitis A., Tsoumanis A., Papadiamantis A., Valsami-Jones E., Lynch I., Melagraki G. (2020). Zeta-potential read-across model utilizing nanodescriptors extracted via the NanoXtract image analysis tool available on the Enalos Nanoinformatics Cloud Platform. Small.

[21] Hunter R.J. (1981). Zeta Potential in Colloid Science: Principles and Applications.

[22] ISO (2012). ISO 13099-1:2012 Colloidal Systems—Methods for Zeta-Potential Determination—Part 1: Electroacoustic and Electrokinetic Phenomena.

[23] ISO (2012). ISO 13099-2:2012 Colloidal Systems—Methods for Zeta-Potential Determination—Part 2: Optical Methods.

[24] ISO (2018). ISO/TR 19997:2018 Guidelines for Good Practices in Zeta-Potential Measurement.

[25] ISO (2014). ISO 13099-3:2014 Colloidal Systems—Methods for Zeta-Potential Determination—Part 3: Acoustic Methods.

[26] Varenne F., Botton J., Merlet C., Vachon J.-J., Geiger S., Infante I.C., Chehimi M.M., Vauthier C. (2015). Standardization and validation of a protocol of zeta potential measurements by electrophoretic light scattering for nanomaterial characterization. Colloids Surf. A.

[27] NIST Standard Reference Material 1980. https://www-s.nist.gov/srmors/certificates/1980.pdf.

[28] Malvern Panalytical Standards. https://www.malvernstore.com/en-us/categories/standards.

[29] ISO (2017). ISO/IEC 17025:2017 General Requirements for the Competence of Testing and Calibration Laboratories.

[30] The Fitness for Purpose of Analytical Methods: A Laboratory Guide to Method Validation and Related Topics. https://eurachem.org/index.php/publications/guides/mv.

[31] ISO (2008). ISO/IEC Guide 98-3:2008 Uncertainty of Measurement—Part 3: Guide to the Expression of Uncertainty in Measurement (GUM:1995).

[32] Ramaye Y., Kestens V., Charoud-Got J., Mazoua S., Auclair G., Cho T.J., Toman B., Hackley V.A., Linsinger T. (2020). The Certification of Electrophoretic Mobility/Zeta Potential of Silica Particles in Aqueous Solution.

[33] Ramaye Y., Kestens V., Charoud-Got J., Mazoua S., Auclair G., Cho T.J., Toman B., Hackley V.A., Linsinger T. (2020). The Certification of Electrophoretic Mobility/Zeta Potential of Silica Particles in Aqueous Solution.

[34] ISO (2015). ISO Guide 30:2015 Reference Materials—Selected Terms and Definitions.

[35] ISO (1998). ISO/TR 3666:1998 Viscosity of Water.

[36] Lide D.R. (2004). Handbook of Chemistry and Physics.

[37] Federer W.T. (1968). Non-negative estimators for components of variance. J. R. Stat. Soc. C.

[38] Comparison of a Measurement Result with a Certified Value. https://ec.europa.eu/jrc/sites/jrcsh/files/erm_application_note_1_en.pdf.

[39] International Vocabulary of Metrology—Basic and General Concepts and Associated Terms (VIM). 3rd ed. JCGM. https://www.bipm.org/en/publications/guides/vim.html.

[40] ISO (2017). ISO 22412:2017 Particle Size Analysis—Dynamic Light Scattering (DLS).

[41] van de Hulst H.C. (1981). Light Scattering by Small Particles.

[42] Linsinger T.P.J., Roebben G., Solans C., Ramsch R. (2011). Reference materials for measuring the size of nanoparticles. Trends Anal. Chem..

[43] Varenne F., Rustique E., Botton J., Coty J.-B., Lanusse G., Lahcen M.A., Rio L., Zandanel C., Lemarchand C., Germain M. (2017). Towards quality assessed characterization of nanomaterial: Transfer of validated protocols for size measurements by dynamic light scattering and evaluation of zeta potential by electrophoretic light scattering. Int. J. Pharm..

[44] Miller J.F. (2020). Determination of protein charge in aqueous solution using electrophoretic light scattering: A critical investigation of the theoretical fundamentals and experimental methodologies. Langmuir.

[45] Strand S.P., Tømmeraas K., Vårum K.M., Østgaard K. (2001). Electrophoretic light scattering studies of chitosans with different degrees of *N*-acetylation. Biomacromolecules.

[46] Kurakula M., Naveen N.R. (2020). In situ gel loaded with chitosan-coated simvastatin nanoparticles: Promising delivery for effective anti-proliferative activity against tongue carcinoma. Mar. Drugs.

[47] Liao D.L., Wu G.S., Liao B.Q. (2009). Zeta potential of shape-controlled TiO_2_ nanoparticles with surfactants. Colloids Surf. A.

[48] Novak S., Lorenzetti M., Drame A., Vidmar J., Ščančar J., Filipič M. (2016). Diversity of TiO2 nanopowders’ characteristics relevant to toxicity testing. J. Nanopart. Res..

