# Supplementary material for: Development and Validation of Optical Methods for Zeta Potential Determination of Silica and Polystyrene Particles in Aqueous Suspensions
Source: Materials (Basel). 2021 Jan 8;14(2):290. doi: 10.3390/ma14020290 (PMC7827561; doi:10.3390/ma14020290)
Supplement: Supplementary file 1 [file materials-14-00290-s001.pdf]

materials

Supplementary Materials

Development and Validation of Optical Methods for Zeta Potential Determination of Silica and Polystyrene Particles in Aqueous Suspensions

Yannic Ramaye [1], Marta Dabrio [1], Gert Roebben [1,2] and Vikram Kestens [1,*]

[1] European Commission, Joint Research Centre (JRC), 2440 Geel, Belgium; yannic.ramaye@ec.europa.eu (Y.R.); marta.dabrio@ec.europa.eu (M.D.); gert.roebben@ec.europa.eu (G.R.)
[2] Present address: European Commission, Directorate-General for Internal Market, Industry, Entrepreneurship and SMEs, 1040 Brussels, Belgium
* Correspondence: vikram.kestens@ec.europa.eu; Tel.: +32-1457-1614

1. Normal probability plots

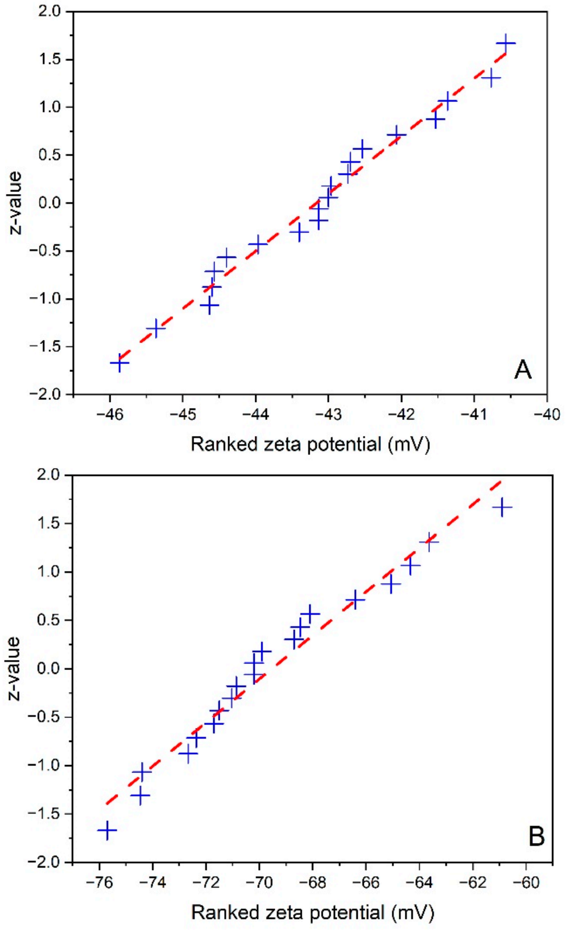

Citation: Ramaye, Y.; Dabrio, M.; Roebben, G.; Kestens, V. Development and Validation of Optical Methods for Zeta Potential Determination of Silica and Polystyrene Particles in Aqueous Suspensions. *Materials* **2021**, *14*, 290. https://doi.org/10.3390/ma14020290

Received: 4 December 2020
Accepted: 5 January 2021
Published: 8 January 2021

Publisher's Note: MDPI stays neutral with regard to jurisdictional claims in published maps and institutional affiliations.

Copyright: © 2021 by the authors. Submitted for possible open access publication under the terms and conditions of the Creative Commons Attribution (CC BY) license (http://creativecommons.org/licenses/by/4.0/).

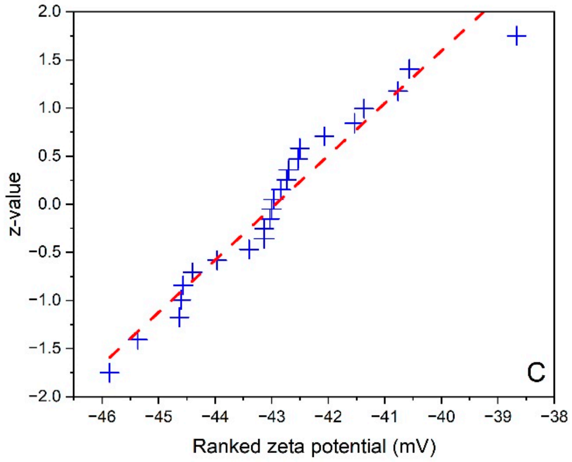

Figure 1. Normal probability plots for zeta potential obtained by ELS for (**A**) DTS 1235 and different cells, (**B**) PS-A and different cells and (**C**) DTS 1235 and different temperatures.
